# Hepatoprotective Effect of Oleuropein-Rich Extract from Olive Leaves against Cadmium-Induced Toxicity in Mice

**DOI:** 10.1155/2020/4398924

**Published:** 2020-04-03

**Authors:** Hedya Jemai, Asma Mahmoudi, Anwar Feryeni, Ines Fki, Zouhaier Bouallagui, Sirine Choura, Mohamed Chamkha, Sami Sayadi

**Affiliations:** ^1^Laboratory of Environmental Bioprocesses, Centre of Biotechnology of Sfax, BP 1177, 3018 Sfax, Tunisia; ^2^Laboratory of Animal Ecophysiology, Faculty of Sciences of Sfax, Tunisia; ^3^Center for Sustainable Development, College of Arts and Sciences, Qatar University, Doha 2713, Qatar

## Abstract

Cadmium (Cd) is a harmful pollutant which mainly affects the liver and kidney. In this work, we investigated the hepatoprotective effects of olive leaf extract based on oleuropein against hepatic cadmium toxicity in mice. Three groups of animals were used: the first one served as the control (C); the second one received intraperitoneal injection of cadmium 2 mg/kg b.w. (CD), administered five times during two weeks; and the third group received the same doses of Cd and simultaneously 16 mg/kg b.w. of oleuropein. Results showed that Cd induced a significant increase in liver injury biomarkers coupled with enhanced lipid peroxidation (MDA) and significant depletion of antioxidants (CAT and SOD). Histological and immunohistochemical analysis confirmed these findings. In fact, we observed a severe central lobular apoptosis and inflammation around central veins. Cotreatment with oleuropein significantly reduced the oxidative damage induced by cadmium. Our findings suggest that oleuropein could be used in the prevention of Cd hepatotoxicity.

## 1. Introduction

Cadmium (Cd) is a nonessential trace element. It is ubiquitous in the environment, a fact that renders it highly toxic to humans and animals [1]. Its toxic effects in the kidneys, heart, vessels, and liver have been widely documented. Moreover, it is highly implicated in cancers [1–3]. Researchers suggest that Cd toxicity is widely related to its induced oxidative stress and apoptosis [4, 5]. In fact, it causes redox homeostasis disruption, coupled with cellular macromolecule oxidation, resulting in DNA and gene expression alteration [6, 7]. In addition, enhanced weakening of physiological antioxidants may also accentuate significantly the Cd-induced oxidative stress [8]. Moreover, it has been reported that Cd can affect some proteins and enzymes by displacing essential metals, mainly zinc and selenium [9]. Consequently, the prevention of Cd toxicity could be based on the use of free radical scavengers and antioxidants [10] such as curcumin [11], catechin, and polyphenols [10, 12]. In fact, many medicinal plants are widely used as a cure of detoxification [13]. Indeed, products from *Olea europaea* leaves and olive oil are regarded as promising sources of bioactive compounds. Nowadays, antioxidants and especially polyphenols come with confirmed protecting features on environmental toxicity in different tissues and organs [14]. In the olive leaves, antioxidant compounds such us oleuropein are present at higher concentrations than in olive oil. Indeed, several studies showed that oleuropein can be regarded as an antioxidant, anti-inflammatory, and antiapoptotic phenolic compound [15–17]. On the other hand, oleuropein protective effects against cadmium were not previously studied in the liver. Nevertheless, it was recently evoked in kidneys, with *in vivo* [18] and *in vitro* studies [19].

Therefore, in this study, we investigate the effects of olive leaf oleuropein against cadmium toxicity in liver tissue of mice.

## 2. Material and Methods

### 2.1. Oleuropein Extraction

**Figure 1:**
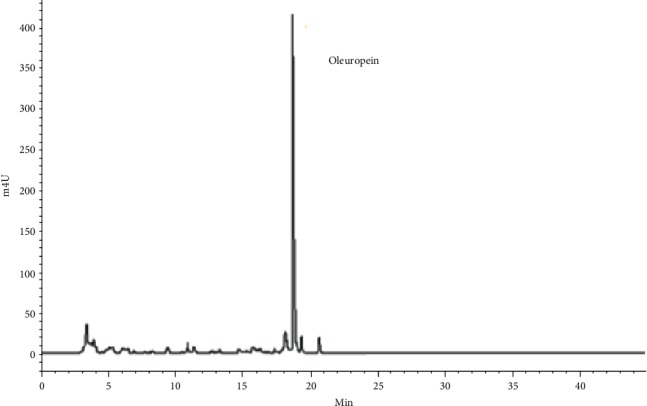
Chromatographic profile of the olive leaf extracts using HPLC at 254 nm.

*Olea europaea* leaves of “Chemlali” variety were used, and the extraction was conducted as described by Mahmoudi et al. [17]. Then, confirmation of oleuropein presence and its quantification was evaluated by high-performance liquid chromatography (HPLC) at 254 nm (Figure 1). The analysis showed that our olive leaf extract was made of oleuropein at 64%.

### 2.2. Experimental Design

Eighteen male mice, with an average 20 ± 3.66 g of body weight, were purchased from the Central Pharmacy of Tunisia. Animal handling conforms to the European Convention (CE. no. 123). The mice were maintained in controlled conditions (photoperiod: 12 h/12 h; temperature: 23 ± 2°C) and had free access to food and water during the one-week period of acclimatization. Then, experiments were conducted for two weeks and based on three groups of mice (*n* = 6):

- Group C served as the control
- Group Cd received intraperitoneal injection of CdCl_2_ divided into five doses of 0.4 mg Cd/kg b.w., during 15 days with an interval of 3 days succeeding to the cumulative dose of 2 mg Cd/kg b.w. The selected dose and protocol of subchronic Cd intoxication was referred from Jemai et al. [5]
- Group Cd+OL received the same dose of Cd in an identical manner with group Cd, with a daily gavage of oleuropein-rich extract succeeding to a final dose of 16 mg/kg b.w.

The selected dose of oleuropein was based on our previous studies [20].

### 2.3. Sample Preparation

After two weeks, we proceeded with the animals' sacrifice to collect blood and organs. We used heparinized tubes for blood collection. Thereafter, centrifugation was applied to separate the plasma (2200 g for 15 min). On the other hand, the liver was detached, weighed, and finally fixed in 10% buffered formalin or stored with serum samples at -80°C.

### 2.4. Serum Biochemical Analysis

A quantitative estimation of aspartate aminotransferase (AST), alanine aminotransferase (ALT), lactate dehydrogenase (LDH), and phosphatase alkaline (PAL) concentrations was conducted by a Vitalab Flexor E automate, at the service of Biochemical Analysis in the CHU Hedi Chaker in Sfax.

### 2.5. Cytosol Preparation from the Liver

A fragment of liver tissue (1 g) was homogenized at 4°C by ULTRA-TURRAX with 10 mL of KCl (1.15%). Then, the homogenate was centrifuged (15 min at 14,000 g) and the supernatant was stockpiled at -80°C for further analysis.

### 2.6. Antioxidant Enzyme Assessment

The SOD activity was evaluated according to Beauchamp and Fridovich [21]. Briefly, the presence of superoxide radicals is revealed by a blue color resulting from its interaction with nitroblue tetrazolium (NBT). SOD acts by removing radicals and, in turn, prevents the formation of formazan blue. Subsequently, the color intensity is inversely proportional to SOD activity determined at 560 nm. On the other hand, the catalase activity assessment was conducted according to the method of Aebi [22] at 620 nm and it was expressed as *μ*M of H_2_O_2_ consumed/min/mg protein.

### 2.7. Total Antioxidant Capacity

The antioxidant capacity in cytosolic samples was determined using the Trolox equivalent. This method is based on the free radical scavenging capacity of the samples, which was calculated as the inhibition percentage of the ABTS+ radical. This inhibition was then equated against a Trolox standard curve, and the results were presented as mM of Trolox equivalents.

### 2.8. Hepatic Lipid Peroxidation Level

The lipid peroxidation in liver cytosol was evaluated via the thiobarbituric acid-reactive substance level accordingly to Park et al. [23]. A mixture of cytosol, distilled H_2_O, SDS, and thiobarbituric acid (TBA) was heated at 95°C for 1 h, which resulted in a colored layer, and then was measured at 532 nm after centrifugation. To determine the TBARS concentration, we used a standard curve based on malondialdehyde (MDA).

### 2.9. Histopathological Exploration

Liver tissues previously fixed in 10% buffered formalin were dehydrated and cleared using ethanol and toluene and then embedded in paraffin. In turn, 5 *μ*m thick sections were prepared and stained with Haematoxylin/Eosin (H&E). Cell necrosis and portal system inflammation were used in the semiquantitative assessment of liver injury. It was based on scores ranging from 0 to 4: (1+) 25% loss, (2+) 50% loss, (3+) 75% loss, and (4+) more than 75% loss. The scores were the result of the examination of 20 fields by section (100x, 400x, and 1000x).

### 2.10. Immunohistochemical Exploration

Liver tissues were heated to retrieve the antigen sites. The sections were then treated with 3% hydrogen peroxide in dH_2_O. Then, they were successively incubated with a blocking solution (1 h at room temperature) and with primary antibodies (overnight at 4°C). The primary antibodies included a rabbit polyclonal antibody against p53, Bcl-2, and COX-2. Thereafter, the reaction was detected with a streptavidin peroxidase Histostain-SP kit. As a result, brown-yellow spots indicated a positive staining which was quantified using the ImageJ software.

### 2.11. Statistical Analysis

GraphPad Prism 6 for Windows was used to analyze the results (mean ± SD). The significance differences were determined by one-way ANOVA and Tukey's multiple comparison post hoc test. The results are shown as follows: vs. control: ^∗∗∗^*p* ≤ 0.001, ^∗∗^*p* ≤ 0.01, and ^∗^*p* ≤ 0.05; vs. Cd: ^+++^*p* ≤ 0.001, ^++^*p* ≤ 0.01, and ^+^*p* ≤ 0.05.

## 3. Results

### 3.1. Body and Liver Weight

After the experimentation period, body and liver weight decreased significantly in cadmium-treated group (Cd), in comparison with the control (*p* < 0.05). Moreover, oleuropein administration in cadmium-treated mice restored significantly the body and liver weight in comparison with the Cd group (*p* < 0.05) (Table 1).

### 3.2. Liver Function Enzyme Activities

**Figure 2:**
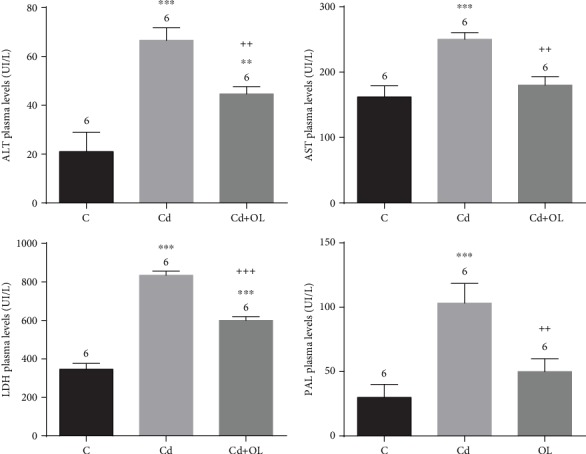
Liver injury biomarkers in the control (C), Cd, and Cd+OL groups.

Biochemical factors in the Cd-intoxicated mice showed significant perturbations. In fact, ALT, AST, LDH, and phosphatase alkaline levels showed a significant increase (*p* < 0.001) compared to the control. The oleuropein administration, in the Cd+OL group, restored significantly all studied parameters (*p* < 0.01) (Figure 2).

### 3.3. Catalase (CAT) and Superoxide Dismutase (SOD) Activities

**Figure 3:**
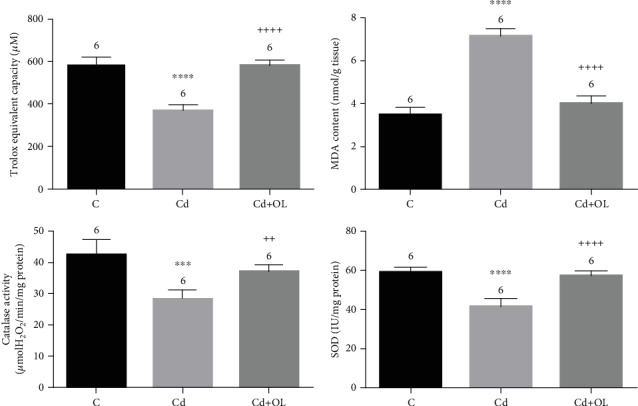
Total antioxidant capacity, lipid peroxidation, and antioxidant enzyme activities in the control (C), Cd, and Cd+OL groups.

The SOD and CAT activities in livers of Cd-intoxicated mice showed a significant decrease compared to those of controls (*p* < 0.001). Moreover, oleuropein-rich extract in Cd-treated mice restored these values with a more pronounced effect in the SOD activity (*p* < 0.001) (Figure 3).

### 3.4. Liver Total Antioxidant Capacity

In Cd-treated mice, the TEAC values showed a significant decrease in liver tissue in comparison with controls (*p* < 0.001). Conversely, oleuropein administration in Cd-treated mice resulted in significant positive effects on the hepatic antioxidant potential (*p* < 0.001) (Figure 3).

### 3.5. Liver TBARS Content

TBARS are the chemical indicators of lipid peroxidation and oxidative stress establishment. The exposure of mice to cadmium engenders a significant increase in the TBARS levels of liver tissue (*p* < 0.001). The simultaneous treatment with cadmium and oleuropein-rich extract significantly inhibited lipid peroxidation of liver tissue, as compared to TBARS values of Cd-treated mice (Figure 3).

### 3.6. Histological Study

**Figure 4:**
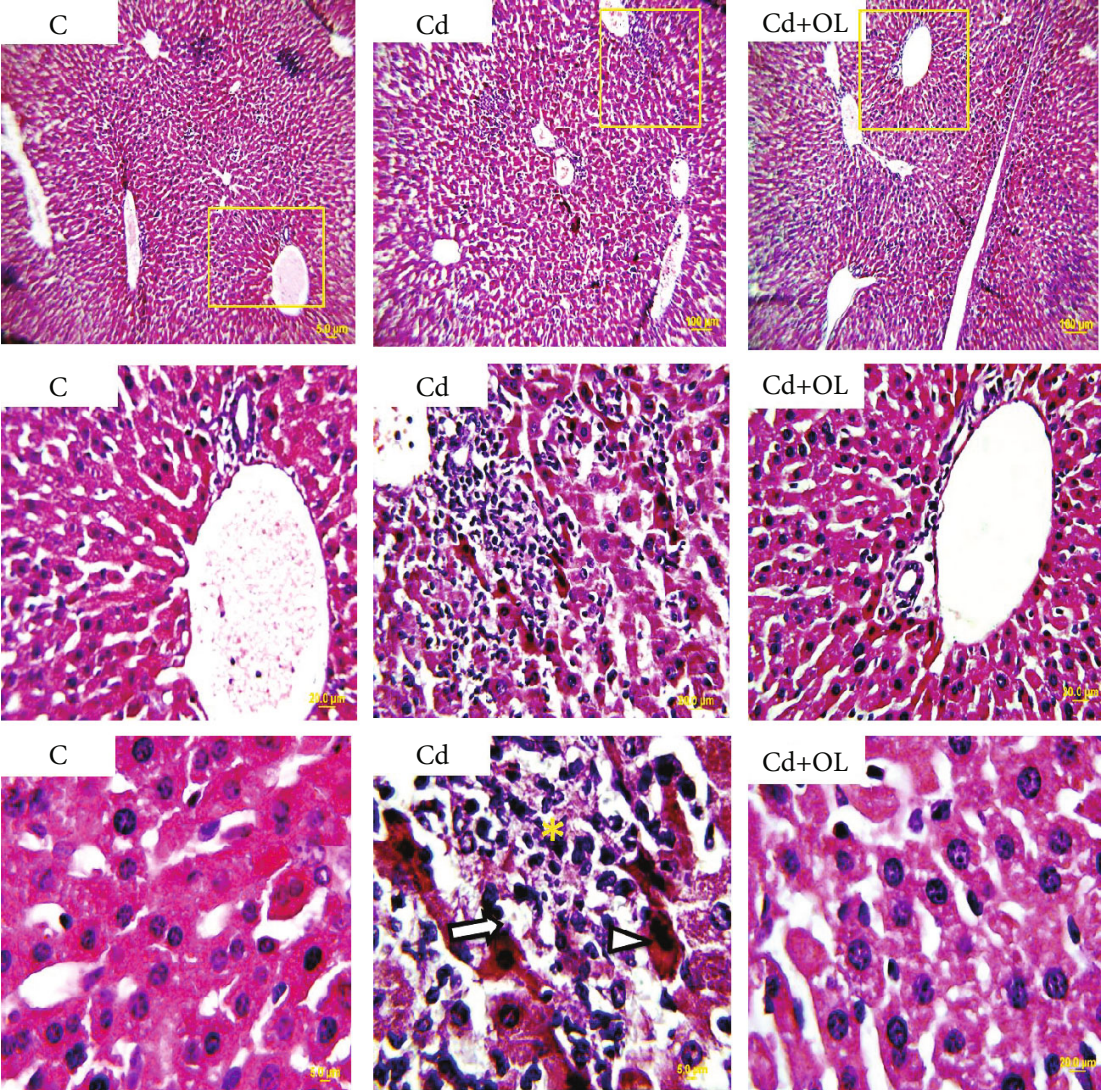
Histological aspect of liver injuries in the control (C), Cd, and Cd+OL groups (scale bar: 5 *μ*m; magnification: 400x). Asterisk: inflammation; arrowhead: monocytes; arrow: necrosis.

The hepatic tissue in the control group presents normal hepatocyte organization (large polygonal cells with eosinophilic cytoplasm and with round nuclei). On the other hand, liver microscopic examination of cadmium-intoxicated mice showed very severe hepatotoxicity with hepatocyte necrosis and leukocyte inflammatory infiltration mainly in the portal triad (Figure 4). All these changes were significantly evaluated by the statistical analysis of histological scoring. Furthermore, a significant increase in the necrosis and inflammation scores was observed in the Cd-treated group. These scores showed a strong reduction in the Cd+OL-treated group for which liver tissues show a similar appearance with those of controls (Table 2).

### 3.7. Immunohistochemistry Analysis

**Figure 5:**
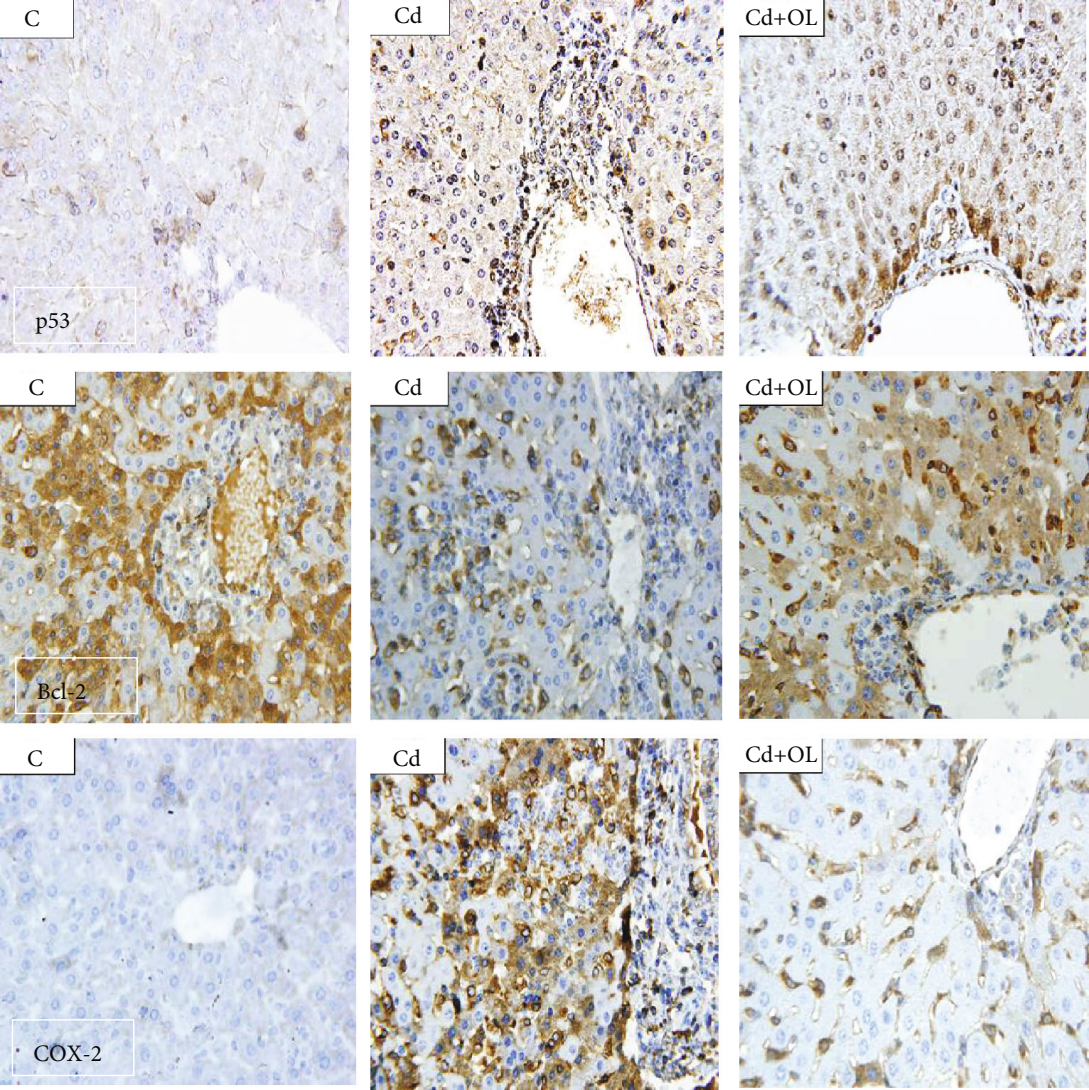
Liver tissue immunohistochemical staining, respectively, with anti-p53, Bcl-2, and COX-2 in the control (C), Cd, and Cd+OL groups (scale bar: 5 *μ*m; magnification: 400x).

The expression of liver p53 in the control showed pale to negative reactions, but it was positively deep in some nucleus of hepatic cells, mainly around the central vein of the Cd-treated group. Moreover, the immunohistochemical expression of p53 was more intense with positive reaction in some cells and with moderate reaction in others. The Cd+OL group showed moderate staining with normal structure of the hepatic cord cells (Figure 5). Indeed, a negative reaction was noticed in the expression of COX-2 in the control group; nevertheless, its expression in the hepatic cells of the Cd-treated group appears with a highly positive one. Furthermore, the expression of COX-2 in the Cd+OL-treated group showed dispersed single positive cells mainly around the central vein (Figure 5). In contrast, protein expression of Bcl-2 of control group livers showed a very high positive reaction. In the Cd group, the immunoexpression of Bcl-2 showed a very faint negative reaction, while it increased in the Cd+OL-treated group (Figure 5). All these changes were significantly evaluated by the statistical analysis of histological scoring (Table 3).

## 4. Discussion

Cd is a prevalent industrial toxic metal. It is a ubiquitous element which can affect the health of humans and animals. Furthermore, Cd engenders oxidative stress in cells and macromolecules and results in severe pathologies and complications [5, 24]. Several studies evoked the Cd toxicity mechanisms which could be related to the lipid peroxidation [9, 25], the DNA expression epigenetic change [26], and the cellular transport pathway inhibition [27]. Moreover, its direct implication in oxidative stress by antioxidant enzyme affection suggests the utility of mineral antioxidants or nutriments such as polyphenols as a protective pathway against Cd toxicity [9]. Indeed, the field of cadmium intoxication therapy has been looking at different phytochemical compounds with hepatoprotective effects [15]. In our previous research [5], the modulatory effects of zinc as a mineral antioxidant against Cd toxicity were established. In this work, the hepatoprotective effects of olive leaf oleuropein were investigated for the first time in response to the Cd intoxication in mice. In fact, oleuropein, because of its confirmed antioxidant potential [16, 17], could have a high positive impact and preventive role in the heavy metal intoxication.

In this study, the body weight in the Cd group significantly decreased. On the other hand, we noticed an increase in relative liver weight. In fact, the alterations of body weight could be due to the dysfunction in the glucocorticoid system in Cd-intoxicated mice. Moreover, it could be explained by the impairment of glucocorticoid hormones which are implicated in glucose, lipid, and protein metabolism [28]. In contrary, the treatment of Cd-intoxicated mice with oleuropein decreased the stunting effects of Cd on body and liver weight.

Our results revealed that a two-week treatment with CdCl_2_ exerts hepatotoxicity. This fact can also be confirmed by the significant increase in serum ALT, AST, ALP, and LDH activities. In fact, these enzymes are considered to be principal indicators of hepatocellular damage. Similar results have been already reported by other researchers [28, 29]. Moreover, the activities of these enzymes are a good indicator of toxicity and reveal the effect of the intoxication period. In our experiments, perturbation was noticed after a two-week intoxication period; however, Haouem and Hani [29] showed that ALT and LDH activities could be revealed with an intoxication period of 8 or 12 weeks.

Several previous studies have confirmed the enhanced activities of these enzymes as a result of Cd intoxication [30]. The hepatotoxicity of Cd injury has been considered to present different mechanisms. It can be the direct effects of Cd by its binding to sulfhydryl groups in the cells or indirectly via inflammation and its mediators, such as cytokines, necrosis, or oxidative stress [1, 5]. Our results demonstrate that oleuropein administration (16 mg/kg b.w.) during a two-week period moderated the hepatotoxicity effects of Cd. In fact, oleuropein engendered a significant decrease in AST, ALT, ALP, and LDH activities compared to the Cd group. This result could be explained by the oleuropein indirect capacities of membrane stabilization, by stopping reactive oxygen species generation and therefore maintaining the membrane structural integrity. This fact was confirmed previously [3].

Cadmium's ability to induce oxidative damage in vital organs, such as the liver, is now confirmed because of its capacity of membrane lipid peroxidation induction via free radicals [31]. Furthermore, it indirectly generates numerous oxygen reactive species, such as superoxide, hydroxyl, and nitric oxide, which possess high deleterious effects on cell membrane leading to its destabilization [25]. Moreover, a significant increase of TBARS in the liver was observed in Cd-intoxicated mice. Administration of oleuropein significantly reversed the Cd-induced peroxidative damage in the liver by lowering TBARS concentrations.

The reduction of lipid peroxidation may be attributed to the antioxidant effect of oleuropein which enhanced the neutralization of free radicals. In fact, oleuropein presents hydroxyl groups in its chemical structure which have the ability to donate hydrogen atom, thereby preventing oxidation. Subsequently, oleuropein with its confirmed capacity to act as a free radical scavenger and metal chelator could significantly prevent cell membranes from Cd to initiate lipid oxidation [32].

Moreover, the main acute damage in cadmium-induced hepatotoxicity is the antioxidant defense system depletion. In fact, Cd affects significantly the antioxidant enzymes which are the first line of defense against free radical damage. Our results show that SOD and CAT antioxidant enzyme activities were significantly reduced in hepatic tissue of Cd-treated mice. The inhibitory action of Cd on SOD is the result of a chemical competition between Cd and Zn or Cu in the enzyme structure, where it creates an inactive form [31, 33]. On the other hand, catalase, which is a hemeprotein, mainly protects cells from H_2_O_2_ and OH oxidative damage [7]. Its significant depletion in the Cd group could be explained by the fact that Cd affects the iron absorption, which is an essential and necessary element for its activity.

Furthermore, our study revealed that oleuropein supplementation in Cd-intoxicated mice significantly increased the antioxidant enzyme activities. This significant observation could be explained by the capacity of oleuropein to reduce free radical accumulation, generated after the lipid peroxidation [9, 14]. Moreover, this hepatoprotective effect of oleuropein could be the result of its biotransformation to hydroxytyrosol, its potential antioxidant metabolite, confirmed to be hepatoprotective in Cd-intoxicated rats [34].

On the other hand, the histopathological study of the liver is in line with our previous results. In fact, we have found in Cd-intoxicated mice an intense hepatotoxicity as proved by hepatic cell necrosis and portal triad leucocyte infiltration. Moreover, the treatment with oleuropein significantly reduced histopathological changes and restored the physiological functions of liver tissue.

Furthermore, with the aim to further elucidate the Cd hepatotoxicity mechanism and cellular protective effect of oleuropein, immunohistochemistry assays, using p53, Bcl-2, and COX-2 antibodies, were performed. In fact, cyclooxygenase-2 (COX-2) is a promoting inflammation protein [35]. In our study, the immunostaining results exhibited an important color intensity in the liver slides of Cd-intoxicated mice compared to controls, evoking a significant hepatic Cd inflammatory effect. However, the immunostaining results in the Cd+OL group was clearly reduced compared to the Cd group, confirming the anti-inflammatory effects of the olive leaf extract based on oleuropein. This finding is in concordance with previous studies confirming the utility of antioxidants in the inflammation inhibition via the tissue COX-2 expression downregulation [14, 36].

Similarly, for p53 expression, which is implicated in apoptosis and its signaling pathways, our results showed an important color intensity in the liver slides of Cd-intoxicated mice, compared to controls and a pale immunostaining in the Cd+OL group. Therefore, Cd-induced toxicity could occur through direct activation of this protein, followed by the initiation of a series of cell death signaling events. Our results are in concordance with previous results confirming the protective effects of oleuropein against tissue hepatotoxicity via the downregulation of the expression of COX-2 and p53 [14, 17]. On the other hand, the expression of Bcl-2 declined in the Cd group liver. In fact, Bcl-2 is an antiapoptotic protein. These results are in agreement with previous data which showed the powerful capability of Cd in increasing p53 and COX-2 expression and reducing Bcl-2 protein expression in hepatic cells [37, 38]. Moreover, Cd inflammation activation could be the direct result of antioxidant depletion such as glutathione and perturbation of the cellular redox status. Additionally, it was confirmed that Cd selectively regulates the expression of inflammatory promoters, such as COX-2 [39]. On the other hand, it was previously confirmed that Cd directly enhanced the tissue p53 accumulation by inhibiting its degradation and thereby promoting apoptosis. This fact is the result of Cd inhibition of the Ube2d family genes [40].

In terms of our immunostaining findings, we concluded that oleuropein-rich extract has the significant potential to protect liver tissue from inflammation and apoptosis which are related to the protein expression regulation [34]. These antiapoptotic, anti-inflammatory, and antioxidant protective effects of the oleuropein-rich extract could make it as an anticancer compound for the liver.

## 5. Conclusion

Our present study showed that oleuropein administration significantly protected hepatic cells in mice by moderating the cadmium-induced toxicity. This powerful hepatoprotection of oleuropein was demonstrated by the significant improvement of the antioxidant activities and by the protein expression regulation of inflammation and apoptosis.

## Figures and Tables

**Table 1:** Body and relative liver weight (g) of mice at the sacrifice day: controls (C), treated with cadmium (Cd), cadmium and oleuropein (Cd+OL).

Parameters	Control	Cd	Cd+OL
Body weight	36 ± 1.88	29±1.76^∗∗^	34 ± 1.15^++^
Relative liver weight	5.13 ± 0.09	7.68±0.07^∗∗^	5.99 ± 0.05^++^

Number of determinations: 18.

**Table 2:** Incidence of histopathological lesions in the liver tissue of the studied groups.

Liver lesions	Control	Cd	Cd+OL
Portal inflammation	0	3	1
Degeneration of hepatocytes	0	3	1
Vacuolar degeneration	0	2	1
Vascular congestion	0	2	1

Injury scores: none (0), mild (1), moderate (2), and severe (3).

**Table 3:** Scoring criteria of immunohistochemistry assay with the specific antibodies.

Staining positive cells			Staining intensity		Final score product	
Groups	Percent (%)	Score 1	Intensity	Score 2	Score 1 × Score 2	Score 3
p53						
C	<5%	0	Absent	0	0-1	0(-)
Cd	51%-75%	3	Strong	3	9-12	3+(+++)
Cd+OL	6%-25%	1	Weak	1	2-4	1+(+)
Bcl-2						
C	51%-75%	3	Strong	3	9-12	3+(+++)
Cd	6%-25%	1	Weak	1	2-4	1+(+)
Cd+OL	26%-50%	2	Moderate	2	5-8	2+(++)
COX-2						
C	<5%	0	Absent	0	0-1	0(-)
Cd	51%-75%	3	Strong	3	9-12	3+(+++)
Cd+OL	6%-25%	1	Weak	1	2-4	1+(+)

## Data Availability

The data used to support the findings of this study are included within this article.

## Additional Points

*Practical Application Abstract*. We investigated the effects of oleuropein-rich extract in hepatic cadmium toxicity in mice. Our results showed that Cd induced hepatic injury, inflammation, and apoptosis with depletion of antioxidants. We validated the positive effects of oleuropein from olive leaf extract, at 16 mg/kg b.w., which considerably reverses all Cd toxicity features. Subsequently, oleuropein could be considerably implicated in the pharmacotherapy of some hepatic cellular and molecular dysfunctions, such as cancer, by its significant regulation of apoptosis and inflammation.
